# Salvage Therapy With Selpercatinib for *RET*-Rearranged NSCLC With Pralsetinib-Related Pneumonitis and Leptomeningeal Disease: A Case Report

**DOI:** 10.1016/j.jtocrr.2023.100581

**Published:** 2023-09-29

**Authors:** Paolo D. d'Arienzo, Niamh Cunningham, Hazel O’Sullivan, Charlotte Grieco, Virjen Patel, Sanjay Popat

**Affiliations:** a Lung Unit, Royal Marsden Hospital, London, United Kingdom; b Clinical Radiology Department, Royal Marsden Hospital, London, United Kingdom; c Division of Clinical Studies, Institute of Cancer Research, London, United Kingdom

**Keywords:** Selpercatinib, Pralsetinib, RET fusion, Leptomeningeal disease, NSCLC, Case Report

## Abstract

Selpercatinib and pralsetinib are RET inhibitors with substantial activity in advanced *RET*-rearranged NSCLC. We present a case of pralsetinib-related pneumonitis and leptomeningeal and brain metastases progression during treatment suspension for pneumonitis. During recovery, selpercatinib administration led to rapid neurologic response and complete intracranial response and allowed pneumonitis resolution. This case supports the safety of selpercatinib in patients with pneumonitis on pralsetinib and highlights its marked efficacy in leptomeningeal disease.

## Introduction

Rearrangements in the RET proto-oncogene occur in 1% to 2% of NSCLCs. Patients are at high risk of intracranial metastases (25% at diagnosis, 46% lifetime risk). Selpercatinib and pralsetinib are tyrosine kinase inhibitors (TKIs) with selective anti-RET activity. Two multicohort phase 1-2 trials reported excellent activity and tolerability in advanced unresectable *RET* fusion-positive NSCLC.^1^^,^^2^ TKI-related pneumonitis manifests as progressive dyspnea with/without cough, fever, and bilateral interstitial or alveolar opacification, in the absence of pulmonary infection or disease progression.^3^ Pralsetinib-related pneumonitis was reported in 12.1% of patients with NSCLC on the ARROW trial,^1^ whereas no cases of selpercatinib-related pneumonitis were reported in patients with NSCLC on the LIBRETTO-001 trial.^2^ TKI switch after TKI-related pneumonitis has been reported in oncogene-addicted NSCLC types,^4^, ^5^, ^6^, ^7^ but not in *RET* fusion-positive NSCLC.

Leptomeningeal disease (LMD), defined as tumor cell spread within the leptomeninges and subarachnoid space, is common in oncogene-addicted NSCLC and associated with poor prognosis. Switch to TKIs with enhanced central nervous system penetration can provide additional benefit. Although both pralsetinib and selpercatinib had good intracranial responses in ARROW and LIBRETTO-001, neither trial enrolled patients with untreated symptomatic intracranial disease. Our case highlights the safety of switching to selpercatinib in a patient intolerant of pralsetinib owing to pneumonitis and its effectiveness in symptomatic progressive LMD.

## Case Presentation

**Figure 1:**
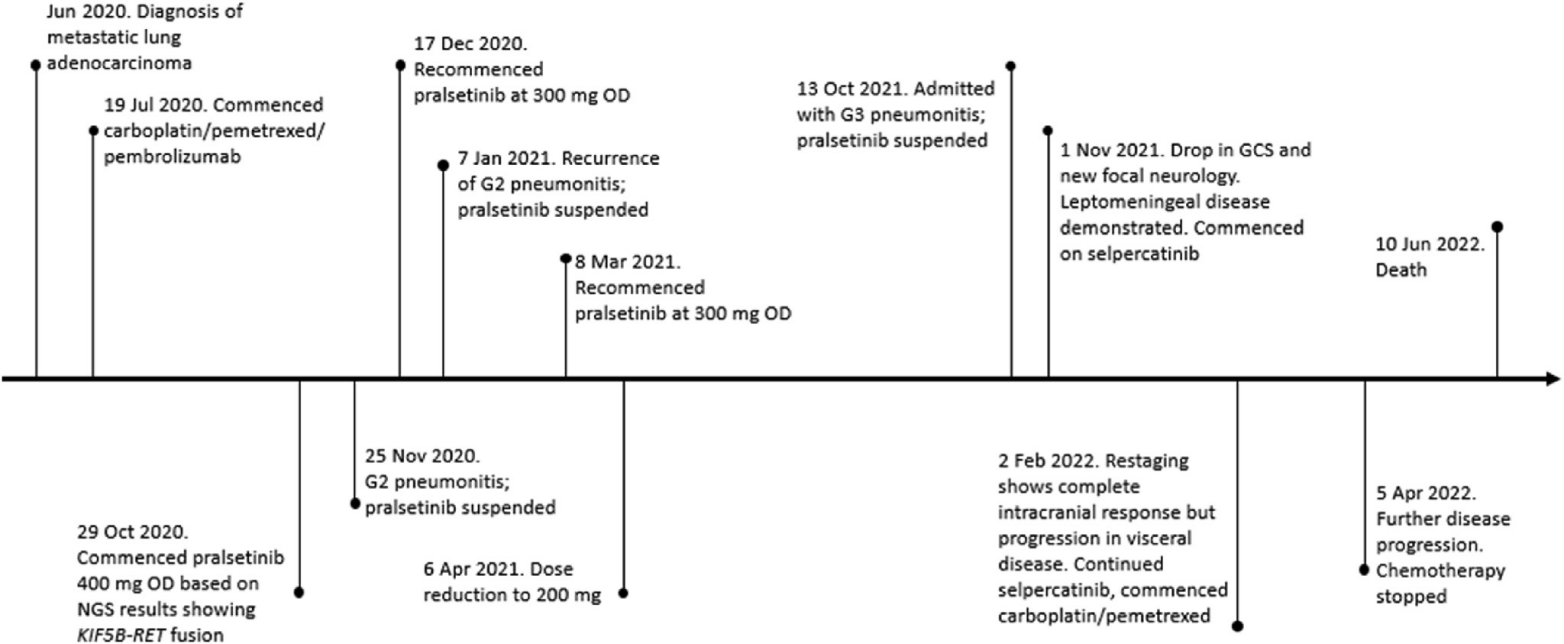
Timeline of events. GCS, Glasgow Coma Scale; Jan, January; Jul, July; Jun, June; Mar, March; NGS, next-generation sequencing; Nov, November; Oct, October; OD, once a day.

A 64-year-old man with adenocarcinoma subtype NSCLC with metastases to the lungs, liver, and brain was admitted in October 2021 with exertional dyspnea and hypoxia. A *KIF5B-RET* fusion was confirmed by RNA-based multitarget next-generation sequencing panel and programmed death-ligand 1 (PD-L1) expression was 35%. Medical history included a transient ischemic attack and 5 pack-year smoking history. Prior treatment included three cycles of carboplatin, pemetrexed, and pembrolizumab to partial response and 12 months of second-line pralsetinib, with partial response. Three small-volume brain metastases (largest lesion 7 mm, left parietal lobe) had been present at baseline and previously responded to pralsetinib without requirement for radiation therapy. Three prior episodes of low-grade (maximum grade 2) pralsetinib-related pneumonitis between November 2020 and April 2021 had been managed with oral steroids, treatment interruption, and dose reduction (400 mg once daily to 200 mg once daily). The sequence of events is detailed in Figure 1.

**Figure 2:**
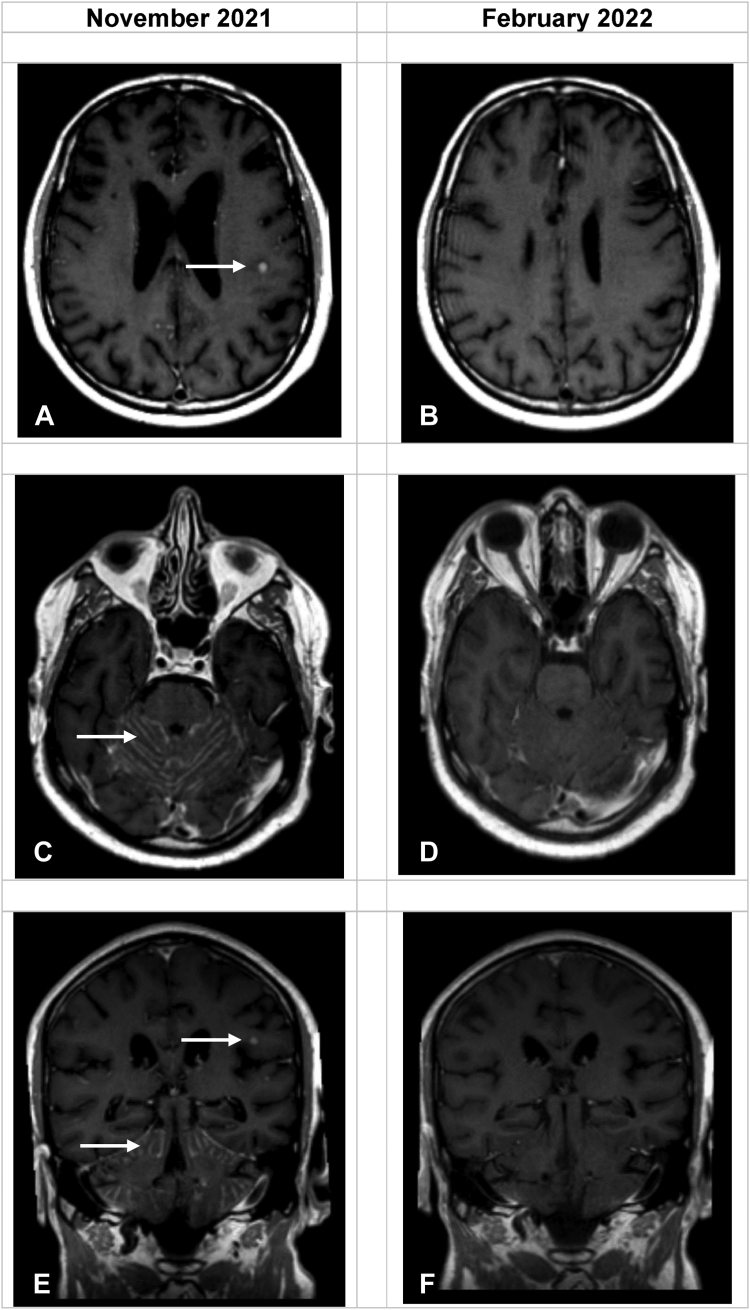
Serial magnetic resonance imaging of the brain. (*A*, *B*) Postcontrast axial T1 sequences at the level of the inferior frontal gyrus. (*C*, *D*) Postcontrast axial T1 sequences at the level of the superior cerebellum. (*E*, *F*) Postcontrast coronal T1 sequences at the level of the occipital horns of the lateral ventricles. The enhancing intra-axial metastasis within the left inferior frontal gyrus and diffuse enhancing leptomeningeal disease of the cerebellum (arrows) have disappeared.

Computed tomography pulmonary angiogram on admission revealed multiple lung metastases and multifocal ground-glass changes consistent with pneumonitis; nil evidence of infection or pulmonary embolus. Neutrophil count, white blood cell count, and C-reactive protein level were within reference range; there were no pyrexia or sputum production. High-flow oxygen and 1 mg/kg oral prednisolone were commenced for treatment of grade 3 drug-related pneumonitis. Pralsetinib was suspended. On day 16 of admission, although still on oxygen and 50 mg prednisolone daily, he became persistently obtunded and developed difficulties in retaining information (Glasgow Coma Scale 13, E3V4M6); performance status dropped to Eastern Cooperative Oncology Group (ECOG) performance status 4. Neurologic examination revealed left upper limb weakness without cranial nerve abnormalities. Magnetic resonance imaging of the brain revealed progression of established brain metastases with new extensive LMD (Fig. 2) confirmed on cerebrospinal fluid cytology. Given the marked neurologic deterioration despite steroids, selpercatinib was commenced at 160 mg twice a day.

**Figure 3:**
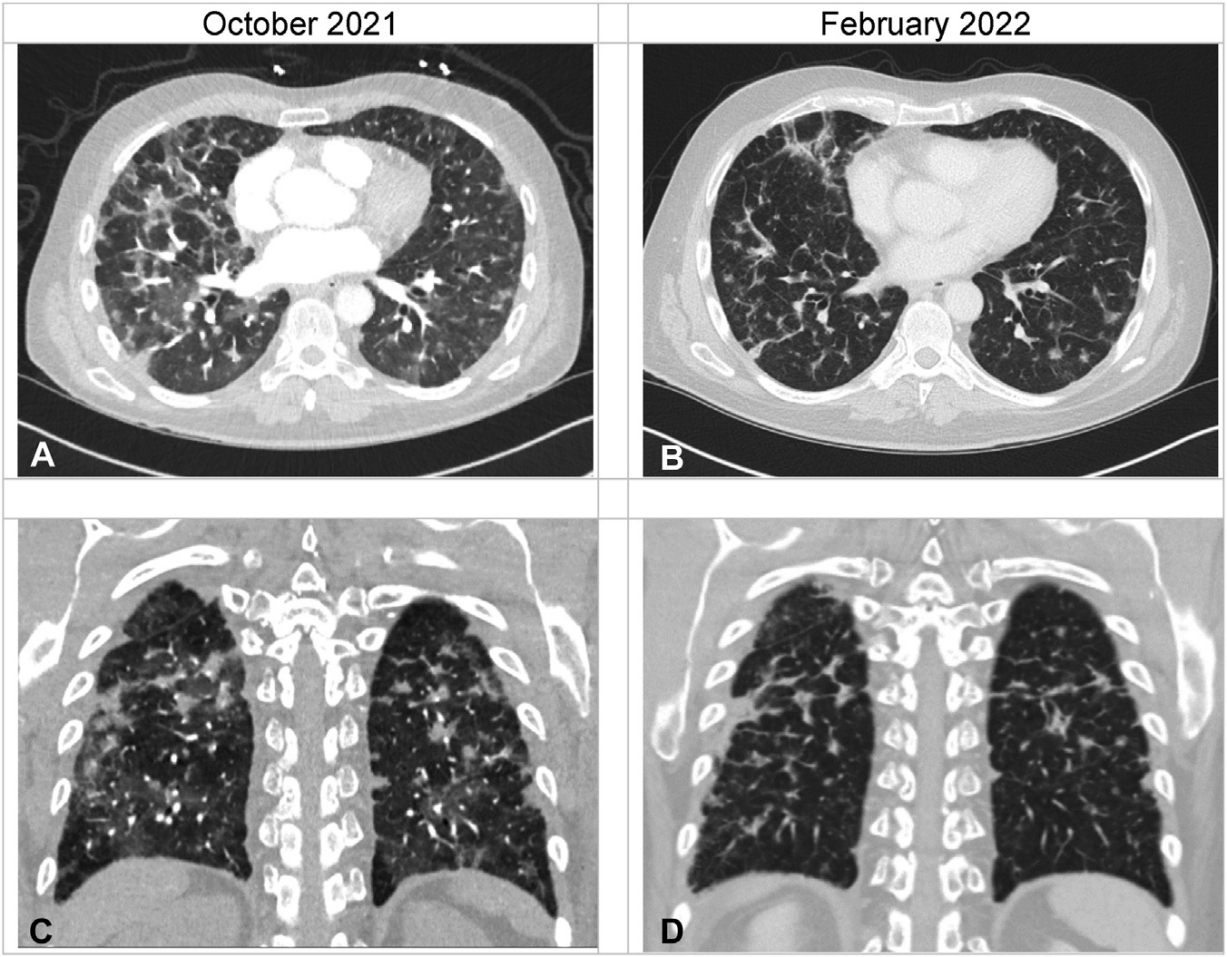
Serial CT imaging of the chest. Panels (*A*) and (*C*) illustrate baseline axial and coronal CT imaging revealing multifocal ground-glass opacification in keeping with pralsetinib-related pneumonitis, which has significantly improved after three months of treatment with selpercatinib (found in panels [*B*] and [*D*]). CT, computed tomography.

The patient had a neurologic response within 72 hours. The abbreviated mental test score went from 7/10 (unable to state date, identify job roles, recall address) to 10/10. Although mild neurologic sequelae, including dysarthria and mild upper limb weakness, persisted, he made a rapid neurologic and respiratory recovery and was discharged on day 35 with ECOG performance status 1, no oxygen requirement, and 20 mg prednisolone daily. Repeat magnetic resonance imaging on the head after three months revealed no residual LMD or brain metastases (Fig. 2*A*–*F*). Restaging computed tomography of the thorax-abdomen revealed resolution of pneumonitic changes (Fig. 3*A*–*D*) but progression in the lungs, liver, and peritoneum. He died five months after despite further carboplatin-pemetrexed-selpercatinib combination treatment.

## Discussion

Our case highlights both the intracranial efficacy and the pulmonary safety of selpercatinib in the setting of leptomeningeal progression on pralsetinib and resolving drug-related pneumonitis.

Two prior case reports described rapid neurologic response to selpercatinib in patients developing LMD while receiving RET-targeting therapy. One case reported partial intracranial response and overall disease control for 10 months after progression on agerafenib (RXDX-105).^8^ The other described partial intracranial response and disease stability for five months after progression on pralsetinib.^9^

Intracranial response to selpercatinib after progression on pralsetinib suggests potential superior intracranial penetration with selpercatinib; however, preclinical and clinical evidence remains inconclusive. In mouse models of intracranial metastasis, pralsetinib was found to have survival benefit at doses inferior to the equivalent recommended human phase 2 dose, and selpercatinib had a relatively low unbound brain-to-plasma partition coefficient (K_p, uu, brain_) at 0.20. Within LIBRETTO-001 and ARROW, both drugs had early and rapid responses in treated and untreated stable brain metastases. Intracranial response rates were 85% for selpercatinib and 70% for pralsetinib.^1^^,^^2^ Nevertheless, relatively few patients had measurable intracranial disease (26 and 10, respectively), and LMD was a trial exclusion.

In our case, given the 2-week treatment interruption owing to pneumonitis, LMD may have represented occult tumor flare rather than acquired pralsetinib resistance. Pralsetinib rechallenge was inappropriate owing to ongoing recovery of grade 3 treatment-related pneumonitis and dose limitations owing to prior toxicity-related dose reductions. Switching to selpercatinib was the only feasible option to attempt intracranial control despite risk of pneumonitis.

In the ARROW trial, 12.1% of patients with NSCLC developed treatment-related pneumonitis, with grade 3 to 4 events in 2.1%. Median onset time was 66 days in treatment-naive patients and 146 days in pretreated patients.^1^

Data on selpercatinib and drug-related pneumonitis are more sparse. The updated analysis of LIBRETTO-001 reported one case of treatment-related pneumonitis in a patient with thyroid cancer.^2^ There were no pneumonitis cases in a real-world cohort of 50 patients with RET fusion-positive NSCLC.^10^ The Food and Drug Administration label included a warning for drug-related pneumonitis and reported a rate of 1.8% for all-grade events; however, the European Medicines Agency did not report pneumonitis as an adverse event.

Several case reports have described safety outcomes for TKI replacement challenge after TKI-related pneumonitis in oncogene-addicted NSCLC types. Erlotinib and afatinib were administered after gefitinib-related pneumonitis,^4^^,^^5^ lorlatinib after alectinib-related pneumonitis,^6^ and tepotinib after capmatinib-related pneumonitis.^7^ Concurrent steroids with TKI switch^4^, ^5^, ^6^ or lower starting TKI doses^4^^,^^5^^,^^7^ were used in some cases. No relapses of TKI-related pneumonitis were reported, suggesting that TKI replacement challenge (with or without steroid cover or lower starting dose) may be safely considered.

Despite complete intracranial response, in our case, extracranial disease control was not achieved, and the patient ultimately passed away. Circulating tumor DNA profiling or tissue biopsy was not feasible to elucidate resistance mechanisms, and next-generation RET inhibitors are still in early phase development. Nevertheless, selpercatinib-related pneumonitis of any grade was not identified, confirming this strategy as a potentially safe and effective method of overcoming pralsetinib-associated pneumonitis and achieving intracranial response, without compromising the RET-kinase inhibitory dose.

## Conclusion

Our case reveals that selpercatinib is safe and effective in patients who develop pralsetinib-related pneumonitis and results in marked intracranial response.

## CRediT Authorship Contribution Statement

**Paolo Davide d’Arienzo:** Conceptualization, Data curation, Writing—original draft.

**Niamh Cunningham:** Writing—review and editing.

**Hazel O’Sullivan:** Writing—review and editing.

**Charlotte Grieco:** Data curation, Writing—review and editing.

**Virjen Patel:** Data curation, Writing—review and editing.

**Sanjay Popat:** Conceptualization, Supervision, Writing—review and editing.
